# Revision of the *Paridris nephta*species group (Hymenoptera, Platygastroidea, Platygastridae)

**DOI:** 10.3897/zookeys.133.1613

**Published:** 2011-10-05

**Authors:** Elijah J. Talamas, Lubomír Masner, Norman F. Johnson

**Affiliations:** 1 Department of Entomology, The Ohio State University, 1315 Kinnear Road, Columbus, Ohio 43212, U.S.A.; 2 Agriculture and Agri-Food Canada, K.W. Neatby Building, Ottawa, Ontario K1A 0C6, Canada; 3 Department of Evolution, Ecology and Organismal Biology, The Ohio State University, 1315 Kinnear Road, Columbus, Ohio 43212, U.S.A.

**Keywords:** Egg-parasitoid, Platygastroidea, key, species description, taxonomic revision

## Abstract

The *Paridris nephta* group is revised (Hymenoptera: Platygastridae). Fifteen species are described, 14 of which are new: *Paridris atrox*Talamas, **sp. n.**(Yunnan Province, China), *Paridris bunun*Talamas, **sp. n.**(Taiwan), *Paridris ferus*Talamas, **sp. n.**(Thailand), *Paridris kagemono*Talamas, **sp. n.**(Japan), *Paridris minator*Talamas, **sp. n.**(Laos, Thailand), *Paridris mystax*Talamas, **sp. n.**(Laos, Thailand), *Paridris nephta*(Kozlov) (Japan, North Korea, South Korea, Far Eastern Russia), *Paridris nilaka*Talamas, **sp. n.**(Thailand), *Paridris reptilis*Talamas, **sp. n.**(Taiwan), *Paridris rugulosus*Talamas, **sp. n.**(Laos, Vietnam), *Paridris solaris*Talamas, **sp. n.**(Laos, Thailand, Vietnam), *Paridris teres*Talamas, **sp. n.**(Vietnam), *Paridris toketoki*Talamas, **sp. n.**(Taiwan), *Paridris verrucosus*Talamas, **sp. n.**(Guangdong Province, China), *Paridris yak*Talamas, **sp. n.**(Thailand).

## Introduction

In 1978, M. Kozlov described a new genus of scelionine wasps based on material from the Russian Far East, with *Tuora nephta* Kozlov as its sole species. No major taxonomic changes occurred in this group until Kononova and Kozlov (2008) treated*Tuora* as a junior synonym of *Paridris*Kieffer, a huge cosmopolitan group. Examination of material from East and Southeast Asia has brought to light many new species that are morphologically close to *Paridris nephta*, constituting a rather homogenous group that may be readily separated from the remainder of *Paridris*.

The goals of this paper are to define the *Paridris nephta* group and describe its species. This work is conducted as part of the Platygastroidea Planetary Biodiversity Inventory and represents a step toward revision of Scelionini sensu lato and resolution of the relationships between its constituent genera. The contributions of the authors are as follows: E.J. Talamas: character definition, species group concept development, species concept development, imaging, key development, manuscript preparation; N.F. Johnson: species concept development, key development, manuscript preparation; L. Masner: species group concept development, manuscript preparation.

## Materials and methods

**Specimens:** This work is based upon specimens deposited in the following collections, with abbreviations used in the text: CNCI, Canadian National Collection of Insects, Ottawa, Canada^1^; IEBR, Institute of Ecology and Biolgical Resources, Hanoi, Vietnam^2^; IZCAS, Chinese Academy of Sciences, Institute of Zoology, Beijing, China^3^; OSUC, C.A. Triplehorn Insect Collection, Columbus, OH^4^; QSBG, Queen Sirikit Botanic Garden, Chiang Mai, Thailand^5^; ROME, Royal Ontario Museum, Ontario, Canada^6^; RMNH, Leiden Nationaal Natuurhistorische Museum, Netherlands^7^.

**Morphology:** Abbreviations and morphological terms used in text: A1, A2, ... A12: antennomere 1, 2, ... 12; claval formula: distribution of the multiporous basiconic sensilla on the underside of apical antennomeres of the female, with the antennomere interval specified followed by the number of sensilla per segment (Bin 1981); palpal formula: number of maxillary and labial palpal segments, respectively; S1, S2, ... S6: metasomal mediosternite 1, 2, ... 6; T1, T2, ... T7: metasomal mediotergite 1, 2, ... 7.; posterior vertex: area between the posterior ocelli and the occipital carina. Morphological terminology largely follows Mikó et al. 2007; the following are illustrated and labeled to facilitate their use.

Axillular carina (axc: Figs 15–16)

Epomial carina (epc; Fig. 7)

Lateral ocellus (loc; Figs 10–11)

Metapleural sulcus (mtps; Fig. 36)

Paracoxal sulcus (pcxs; Fig. 36)

Transverse carina of T2 (trc; Fig. 12)

Transverse pronotal carina (tpc; Fig. 7)

Morphological terms used in this revision were matched to the Hymenoptera Anatomy Ontology (HAO, Yoder et al. 2010) (Appendix I). Identifiers (URIs) in the format http://purl.obolibrary.org/obo/HAO_XXXXXXX represent anatomical concepts in HAO version http://purl.obolibrary.org/obo/hao/2011-05-18/hao.owl. They are provided to enable readers to confirm their understanding of the anatomical structures being referenced. To find out more about a given structure, including, images, references, and other metadata, use the identifier as a web-link, or use the HAO:XXXXXXX (note colon replaces underscore) as a search term at http://glossary.hymao.org. Notable changes in term usage from a previous taxonomic work (Talamas et al. 2011) are given in Appendix I.

The description of surface sculpture is presented in two formats. Areas of the exoskeleton in which the sculptural elements are inseparable are described simply as “sculpture”. For areas in which the sculptural elements vary independently, sculpture is divided into three categories: punctation: round depressions associated with setae; macrosculpture: raised or sunken patterns of texture that are oriented linearly or radially with respect to punctation or the axes of the body; microsculpture: unoriented, very fine wrinkles or pustulations that occur on, in, or between elements of macrosculpture and punctation.

**Information Management:** The locality data reported for primary types are not literal transcriptions of the labels: some abbreviations are expanded; additional data from the collectors are also included. The holotypes should be unambiguously identifiable by means of the unique identifier or the red holotype label. The numbers prefixed with “OSUC ” and “CASENT ” are unique identifiers for the individual specimens (note the blank space after the acronyms). Details on the data associated with these specimens may be accessed at the following link, purl.oclc.org/NET/hymenoptera/hol, and entering the identifier in the form. This monograph also features simultaneous publication and distribution of taxonomic and occurrence records through the Global Biodiversity Information Facility (GBIF) using DarwinCore Archives as in Talamas et al. (2011). All new species have been prospectively registered with Zoobank (Polaszek et al. 2005) and other taxonomic names have been retrospectively registered therein. All names are also registered in the Hymenoptera Name Server (hns.osu.edu). Life sciences identifiers, lsids, may be resolved at the URLs specified in the footnotes or at lsid.tdwg.org.

**Cybertools:**The species descriptions are generated by a database application, vSysLab (purl.oclc.org/NET/hymenoptera/vSysLab), designed to facilitate the generation of taxon by character data matrices, to integrate these with the existing taxonomic and specimen-level database, and to export the data both as text and as input files for other applications. The output is in the format of “Character: Character state(s).”

**Imaging:** Images were produced using Combine ZP and AutoMontage extended-focus software. The individual images are archived at the image database at The Ohio State University (purl.oclc.org/NET/hymenoptera/specimage) and with MorphBank (www.morphbank.net). The latter also contains collections of images organized by plate.

**Species Concept:** For the purpose of this revision, species are defined as taxa diagnosable by putative autapomorphies or a unique combination of fixed character states.

## Comments on ParidrisKieffer

The genus *Idris* was described by Arnold Förster in 1856, and the name has been used as the root for a number of generic names in Platygastroidea. Wheeler (1935) proposed that it would be a useful root for names within the Formicidae, relieving the stress on roots such as –*myrmex* and –*myrma*. According to Wheeler, the name is a substantive noun, derived from classical Greek, meaning “the knowing or provident one.” As such, it may be either masculine or feminine in grammatical gender. While workers in Platygastroidea have treated the name and its derivatives as masculine, myrmecologists have used names with this root as feminine nouns. Here, we continue our tradition and use *Paridris* as a masculine noun.

The Nearctic *Paridris brevipennis* Fouts has one documented host association with *Gryllus pennsylvanicus* Burmeister (label data of a specimen in the USNM reported by Masner and Muesebeck 1968). Based on this information, we speculate that the species of the *Paridris nephta* group are also parasitoids of gryllid eggs.

With the exception of Masner (1976), previous workers treated *Paridris* within only a restricted geographical context (Mani and Sharma 1982, Galloway and Austin 1984, Kozlov and Kononova 1985, Kozlov and Kononova 1990, Kononova and Petrov 2000, Lê 2000, Mineo 2005, Rajmohana 2006, Kononova and Kozlov 2008). Perhaps unsurprisingly, the characters they used for identification of the genus are insufficient when the world fauna is considered: the length of R1 (postmarginal vein) is variable; the shape of the metascutellum is highly variable, and in females may be entirely obscured by the horn of T1; the lateral ocellus is often close to the inner orbit of the compound eye; and the horn of T1 is missing in some members of the *Paridris nephta* species group.

Previous authors have mentioned that *Paridris* may be confused with *Probaryconus* (Galloway and Austin 1984) and *Anteris* Förster (Masner 1976). Masner (1976) indicated that *Anteris* and Neotropical *Paridris* are close to each other, and indeed they are highly similar in most of the external characters typically used for identification. Based on a yet unpublished phylogeny, we consider many of the similarities between these two genera to be convergent and not indicative of close relationship.

Separation of *Paridris* from *Probaryconus* is a more complicated matter because both are polytypic. *Probaryconus* has neither notauli (Figs 9–10) nor an externally developed metascutellum (Figs 9–10), and always has spines, points, or dense tufts of setae on the propodeum (Figs 9–10). The epomial carina (Fig. 7) is present in *Probaryconus* (always absent in *Paridris*), with the exception of one widespread species group (Fig. 8) that also has setose eyes and a strongly reduced postmarginal vein. The transverse carina of T2 (Fig. 12) unambiguously identifies *Paridris* but is not present in all species (e.g. the *Paridris nephta species* group). In some Neotropical and Oceanic species of *Paridris*, the lateral propodeal carinae form two points lateral to the metasomal depression, similar to the propodeal points in *Probaryconus* Kieffer. The following key separates *Probaryconus* and *Anteris* from *Paridris* with the fewest characters possible.

### Key to separate Paridris, Probaryconus and Anteris

**Table:** 

1	Palpal formula 2-1 (Fig. 1); female T7+8, when extruded with ovipositor, connected to T6 by short, unsegmented conjunctiva (Fig. 3)	*Anteris*
–	Palpal formula 4-2 (Fig. 2); female T7+8, when extruded with ovipositor, connected to T6 by long, segmented conjunctiva (Fig. 4)	2
2	Metanotum visible medially and unaltered by horn of T1, or horn absent (Figs 10, 12)	3
–	Metanotum obscured medially by horn of T1 (Figs 9, 11)	4
3	Metascutellum visible externally, shape variable (Fig. 12)	*Paridris*
–	Metascutellum not visible externally (Fig. 10)	*Probaryconus*
4	Lateral ocellus remote from inner orbit, separated by distance of at least one ocellar diameter (Fig. 11)	*Paridris*
–	Lateral ocellus contiguous with inner orbit or separated by distance less than one ocellar diameter (Fig. 10)	*Probaryconus*

### Diagnosis of nephta species group

The *Paridris nephta* species group can be separated from the remainder of *Paridris* by the combination of the following characters: occipital carina reaching base of mandible; mesoscutal suprahumeral sulcus absent mesal to notaulus; scutoscutellar and posterior scutellar sulci comprised of deep cells; metascutellum bispinose, glabrous; mesepisternum below femoral groove with coarse rugose sculpture; paracoxal and metapleural sulci not fused in dorsal half of metapleuron (Fig. 32); posterior margin of metapleuron with triangular point above metapleural sulcus; propodeum coarsely punctate rugose; plica indistinguishable or poorly distinguished from background sculpture of propodeum; anterior T2 without transverse carina; T6 evenly rounded, without dense microsculpture; felt field on S2 punctate, present throughout length of sternite.

Sexual dimorphism combined with the small number of males prevented us from associating males with females for all but two species, *Paridris mystax* and *Paridris nephta*. Consequently, only females are treated in the key and descriptions. Males for *Paridris mystax* and *Paridris nephta* have been entered as determined material, but not as paratypes for *Paridris mystax*. Four other male morphotypes have been imaged and can be found online at www.specimage.osu.edu and www.morphbank.net^8,9,10,11^.

**Figure 1–6.**
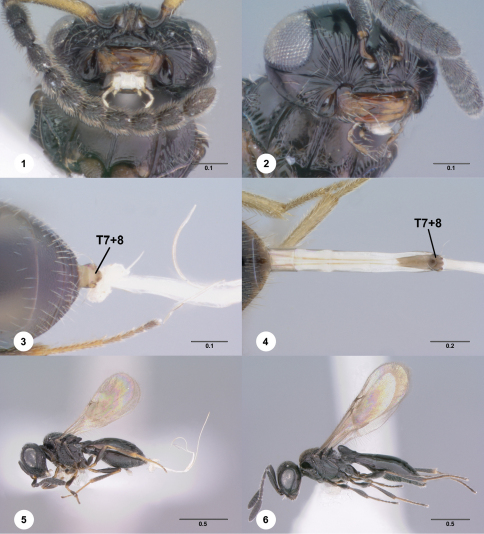
**^62^** **1** *Anteris* sp., head, mouthparts, ventral view, male (OSUC 241115)**2** *Paridris* sp., head, mouthparts, anteroventral view, female (OSUC 190976)**3** *Anteris*sp., T5–T7, ovipositor, female (OSUC 261917)**4** *Paridris nilaka*, T6–T7, ovipositor, female (OSUC 266165)**5** *Anteris*sp., lateral habitus, female (OSUC 261917)**6** *Paridris*sp., lateral habitus, female (OSUC 191490). Scale bar in millimeters.

**Figures 7–12.**
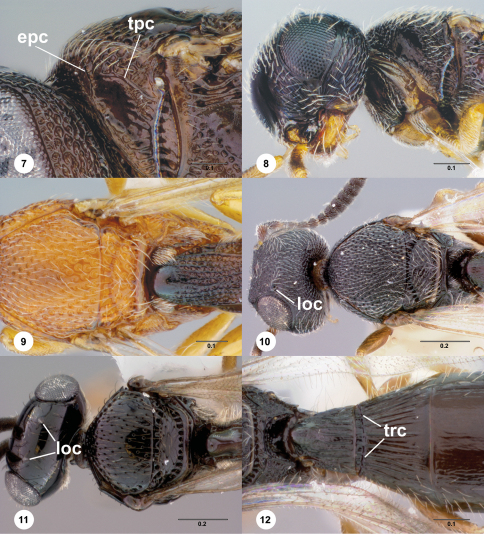
**^63^** **7***Probaryconus*sp., pronotum, lateral view, female (OSUC 146809)**8** *Probaryconus*sp., head and pronotum, female (OSUC 58741)**9** *Probaryconus*sp., mesosoma and T1, dorsal view, female (OSUC 404933)**10** *Probaryconus rufipes* (Kieffer), head and mesosoma, dorsal view, female (OSUC 396820)**11** *Paridris*sp., head, mesosoma, T1, dorsal view, female (OSUC 262120)**12** *Paridris*sp., metascutellum, propodeum, T1–T2, dorsal view, female (OSUC 265183). Scale bar in millimeters.

### Key to females of the Paridris nephta species group (a Lucid key is included as a Appendix II).

**Table:** 

1	Brachypterous, forewing not reaching apex of metasoma in repose (Figs 31, 33, 67, 69)	2
–	Macropterous, forewing extending beyond apex of metasoma in repose	3
2	A7 with basiconic sensillum (Fig. 14); sculpture of T3 reduced medially (Fig. 33); metapleural sulcus simple dorsally (Fig. 36)	*Paridris ferus* Talamas, sp. n.
–	A7 without basiconic sensillum (Fig. 13); sculpture of T3 not reduced medially (Fig. 72); metapleural sulcus foveolate dorsally (Fig. 68)	*Paridris reptilis* Talamas, sp. n.
3	Ventral clypeal margin edentate (Fig. 89); T3 covered in finely reticulate microsculpture (Fig. 90)	*Paridris teres*Talamas, sp. n.
–	Ventral clypeal margin serrate (Figs 53, 101, 107); sculpture of T3 variable	4
4	Ventral metapleural area entirely setose (Fig. 50); frons densely setose ventrolaterally (Fig. 53); head and metasoma black (Fig. 49)	*Paridris mystax* Talamas, sp. n.
–	Ventral metapleural area with glabrous area (Fig. 62); frons moderately to sparsely setose ventrolaterally (Fig. 107); body color variable	5
5	Notaulus absent or indicated only at posterior margin of mesoscutum (Figs 22, 106)	6
–	Notaulus present through posterior half of mesoscutum, usually reaching mesoscutal suprahumeral sulcus as smooth furrow or row of punctures (Figs 58, 76)	8
6	T1–T5, S3 with microsculpture throughout (Figs 17, 102); T3 evenly reticulate in medial third (Fig. 102)	*Paridris verrucosus* Talamas, sp. n.
–	Metasoma without microsculpture (Figs 18, 105); T3 longitudinally strigose (Figs 12, 105)	7
7	Frons immediately below median ocellus smooth (Fig. 23); axillular carina rounded dorsally (Fig. 16); S3 with longitudinal striae (Fig. 18)	*Paridris atrox* Talamas, sp. n.
–	Frons immediately below median ocellus rugose, with setigerous foveae (Fig. 107); axillular carina pointed dorsally (Fig. 15); S3 without longitudinal striae	*Paridris yak*Talamas, sp. n.
8	A7 with basiconic sensillum (Fig. 14)	9
–	A7 without basiconic sensillum (Fig. 13)	10
9	R1 (postmarginal vein) distinctly shorter than r-rs (stigmal vein) (Fig. 109); T3 smooth with weakly impressed longitudinal striae laterally (Fig. 30), microsculpture absent; punctation of head fine (Fig. 29)	*Paridris bunun* Talamas, sp. n.
–	R1 (postmarginal vein) about as long as r-rs (stigmal vein) (Fig. 111); T3 with prominent longitudinal strigae laterally, often strigose throughout, microsculpture usually present (Fig. 48); punctation of head variable, often coarse (Fig. 47)	*Paridris minator* Talamas, sp. n.
10	Mesoscutellum punctate, interspaces between punctures smooth and usually broad (Figs. 58, 82)	11
–	Mesoscutellum rugulose to areolate (Figs 40, 63, 76, 94)	12
11	Frons evenly striate throughout, striae directly above interantennal process sometimes effaced (Fig. 59); interstitial punctation on frons very fine (Fig. 59)	*Paridris nephta* (Kozlov)
–	Frons directly below median ocellus coarsely strigose to rugose; frons always with smooth area above interantennal process; interstitial punctation on frons coarse (Fig. 83)	*Paridris solaris* Talamas, sp. n.
12	Frons evenly striate throughout, with microsculpture present interstitially (Fig. 41)	*Paridris kagemono* Talamas, sp. n.
–	Striae of frons, if present, not uniform throughout, microsculpture absent (Figs 65, 77, 95)	13
13	Pronotum with uniform, fine, white setae along transverse pronotal carina (Fig. 66); body dark brown to black (Fig. 61)	*Paridris nilaka* Talamas, sp. n.
–	Pronotum with dark, bristlelike setae along transverse pronotal carina (Figs 77, 94); body color variable	14
14	Pronotum below transverse pronotal carina mostly smooth, with sparse rugulae (Fig. 77)	*Paridris rugulosus* Talamas, sp. n.
–	Pronotum below transverse pronotal carina densely punctate (as in Fig. 66)	*Paridris toketoki* Talamas, sp. n.

#### Paridris atrox

Talamas sp. n.

urn:lsid:zoobank.org:pub:BCD3BD6E-5E29-447F-AEB6-176C19EEF3E8

urn:lsid:biosci.ohio-state.edu:osuc_concepts:275737

http://species-id.net/wiki/Paridris_atrox

Figures 16 19–24 Morphbank^12^ 

##### Description.

Female body length: 2.73 mm (n=1). Color of head: reddish brown. Ventral clypeal margin: serrate. Sculpture of frons medially: smooth. Sculpture of frons immediately ventral of median ocellus: dorsoventrally strigose laterally. Microsculpture of frons: absent. Sculpture of posterior vertex: irregularly rugulose. Sculpture of gena: irregularly rugulose. Basiconic sensillum on A7: absent.

Wings: macropterous, apex of forewing extending beyond posterior margin of T3. Length of R1: equal to r-rs. Notaulus: absent. Color of mesosoma: variably orange to brown. Sculpture of mesoscutum medially: areolate rugulose. Sculpture of mesoscutellum: areolate rugulose. Dark bristlelike setae along transverse pronotal carina: present. Sculpture ventral of transverse pronotal carina: rugulose posteriorly. Sculpture of femoral groove: striate below mesopleural pit. Sculpture of ventral half of posterior mesepimeral area: rugulose. Fine setigerous punctures on dorsal half of posterior mesepimeral area: absent. Mesopleural carina: present. Setation of ventral metapleural area: absent in area immediately below metapleural sulcus. Setation of metapleural triangle: moderately dense. Color of legs: yellow throughout.

Color of metasoma: reddish brown. Horn of T1: bulge smooth, at least anteriorly. Microsculpture of T2: absent. Microsculpture on T3: absent. Macrosculpture of T3 medially: weakly longitudinally strigose. Macrosculpture of T3 laterally: longitudinally strigose. Microsculpture of T4: absent. Macrosculpture of T4 laterally: weakly rugulose. Punctation of T4: moderately dense throughout. Macrosculpture of T5: absent. Punctation of T5: moderately dense throughout. Microsculpture of S3: absent. Macrosculpture of S3 laterally: weakly longitudinally strigose.

**Figures 13–18.**
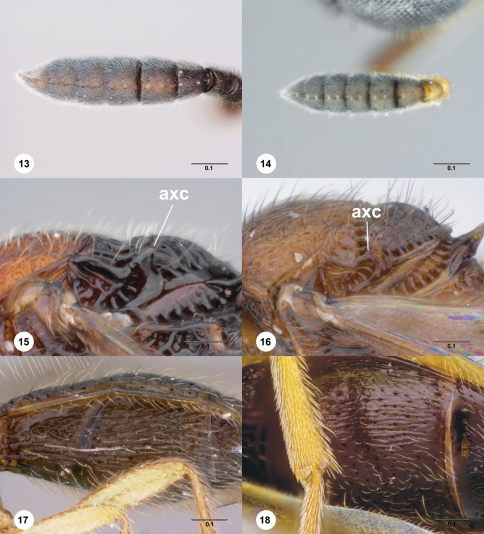
**^64^** **13** *Paridris nilaka*, sp. n., antennal clava, ventral view, female (OSUC 334247)**14** *Paridris minator*, sp. n., antennal clava, ventral view, female holotype (OSUC 237531)**15** *Paridris yak*,sp. n, scuto-axillar complex, lateral view, female holotype (OSUC 237530)**16** *Paridris atrox*, sp. n., scuto-axillar complex, lateral view, female holotype (OSUC 241473)**17** *Paridris verrucosus*, sp. n., S2–S3, ventrolateral view, female holotype (OSUC 334249)**18** *Paridris atrox*, sp. n., S3, ventrolateral view, female holotype (OSUC 241473). Scale bars in millimeters.

**Figures 19–24.**
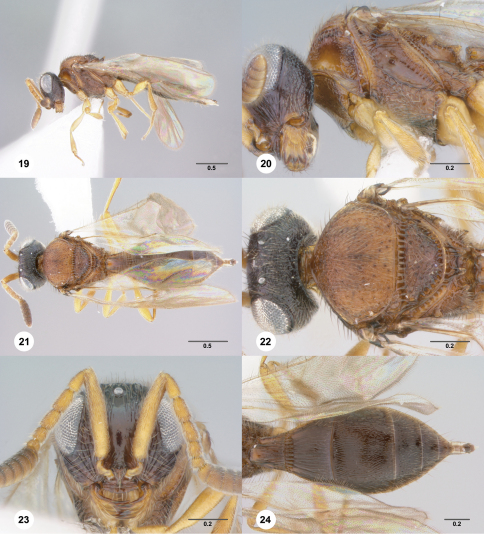
**^65^***Paridris atrox* sp. n., female holotype (OSUC 241473) **19** Lateral habitus**20** Head and mesosoma, lateral view**21** Dorsal habitus**22** Head and mesosoma, dorsal view**23** Head, anterior view**24** Metasoma, dorsal view. Scale bars in millimeters.

##### Diagnosis.

 *Paridris atrox* may be separated from the other members of the *Paridris nephta* species group by the absence of notauli and the presence of striation on S3.

##### Etymology.

 *Paridris atrox* is named for the severe appearance of its head, its mandibles in particular. The specific epithet is adjectival, and means “fearsome” in Latin.

##### Link to Distribution Map.

^13^

##### Material Examined.

Holotype, female: **CHINA:** Yunnan Prov., Baoshan City, 28 km (air)SE Tengyue (Teng Chong), pass over Gaoligong Mts., clearing / natural forest, Luoshuidong, 24°57'N, 98°45'E, 2300m, 26.X–31.X.1998, flight intercept trap, C. Griswold, D. Kavanaugh & C. L. Long, OSUC 241473 (deposited in IZCAS).

#### Paridris bunun

Talamas sp. n.

urn:lsid:zoobank.org:act:A3CA8ADB-0B8F-47C1-A757-9CEAE44779A2

urn:lsid:biosci.ohio-state.edu:osuc_concepts:273886

http://species-id.net/wiki/Paridris_bunun

**Figures 25–30.**
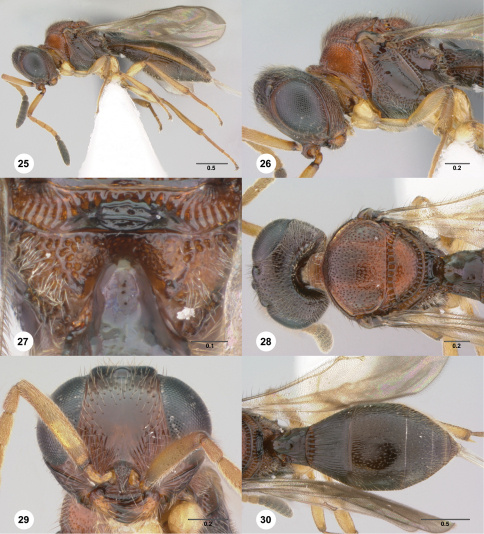
**^66^***Paridris bunun* sp. n., female holotype (OSUC 262237) **25** Lateral habitus**26** Head and mesosoma, lateral view**27** Propodeum, posterodorsal view**28** Head and mesosoma, dorsal view**29** Head, anterior view**30** Metasoma, dorsal view. Scale bars in millimeters.

Figures 25–30 Morphbank^14^ 

##### Description.

 Female body length: 3.41 mm (n=1). Color of head: dark red, becoming darker dorsally. Ventral clypeal margin: serrate. Sculpture of frons medially: smooth. Sculpture of frons immediately ventral of median ocellus: dorsoventrally strigose laterally. Microsculpture of frons: absent. Sculpture of posterior vertex: finely punctate. Sculpture of gena: densely and finely punctate. Basiconic sensillum on A7: present.

Wings: macropterous, apex of forewing extending beyond posterior margin of T3. Length of R1: less than r-rs. Notaulus: present in posterior half of mesoscutum. Color of mesosoma: variably red to black. Sculpture of mesoscutum medially: densely punctate throughout. Sculpture of mesoscutellum: densely punctate. Dark bristlelike setae along transverse pronotal carina: absent. Sculpture ventral of transverse pronotal carina: finely punctate. Sculpture of femoral groove: smooth. Sculpture of ventral half of posterior mesepimeral area: smooth. Fine setigerous punctures on dorsal half of posterior mesepimeral area: present. Mesopleural carina: present along anterior half of femoral groove. Setation of ventral metapleural area: absent in area immediately below metapleural sulcus. Setation of metapleural triangle: moderately dense. Color of legs: yellow throughout.

Color of metasoma: reddish brown. Horn of T1: bulge smooth, at least anteriorly. Microsculpture of T2: absent. Microsculpture on T3: absent. Macrosculpture of T3 medially: absent. Macrosculpture of T3 laterally: weakly longitudinally strigose. Microsculpture of T4: absent. Macrosculpture of T4 laterally: weakly longitudinally strigose. Punctation of T4: sparse in medial third, moderately dense laterally. Macrosculpture of T5: absent. Punctation of T5: absent in medial third, moderately dense laterally. Microsculpture of S3: absent. Macrosculpture of S3 laterally: absent.

##### Diagnosis.

 *Paridris bunun* is most similar to *Paridris minator*, though the two have widely disjunct distributions, Taiwan and Southeast Asia, respectively. The two may be separated by the medially smooth T3 and short R1 (postmarginal vein) of *Paridris bunun* and the longer setation of the body in *Paridris minator*. *Paridris bunun* is a much larger species than *Paridris minator*, but it is known from a single specimen and thus we are not able to assess its size variation. Some species of the *Paridris nephta* group are known to exhibit significant size variation (e.g. *Paridris nilaka*) and thus size should be used cautiously.

##### Etymology.

 The species is named for the Bunun tribe of Taiwan that historically occupied the region where it was collected. The name is treated as a noun in apposition.

##### Link to Distribution Map.

^15^

##### Material Examined.

Holotype, female: **TAIWAN:** Taiwan Prov., Pingtung Co., T’eng-chih (Tengchi) Medium-Altitude Experiment Station, 23°05.75'N, 120°47.37'E , 1660m, 3.VI–5.VI.2008, yellow pan trap, L. Masner, OSUC 262237 (deposited in CNCI).

#### Paridris ferus

Talamas sp. n.

urn:lsid:zoobank.org:act:6B7296C1-4E71-4A35-B937-ABB1949B7DBE

urn:lsid:biosci.ohio-state.edu:osuc_concepts:241281

http://species-id.net/wiki/Paridris_ferus

**Figures 31–36.**
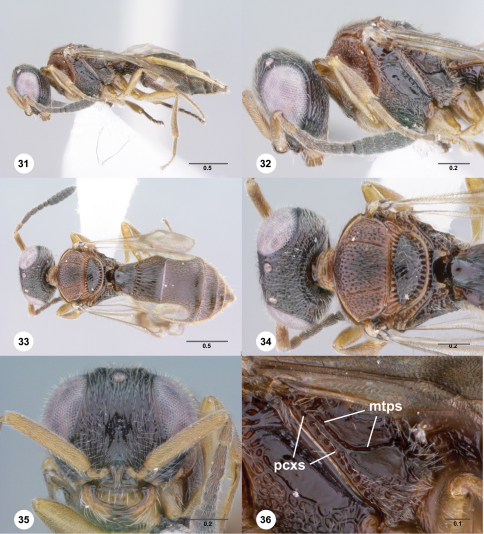
**^67^***Paridris ferus* sp. n., female holotype (OSUC 192426) **31** Lateral habitus**32** Head and mesosoma, lateral view**33** Dorsal habitus**34** Head and mesosoma, dorsal view**35** Head, anterior view**36** Metapleuron, lateral view. Scale bars in millimeters.

Figures 31–36 Morphbank^16^ 

##### Description.

Female body length: 2.89 mm (n=1). Color of head: black throughout. Ventral clypeal margin: serrate. Sculpture of frons medially: smooth. Sculpture of frons immediately ventral of median ocellus: dorsoventrally striate throughout. Microsculpture of frons: absent. Sculpture of posterior vertex: punctate rugulose. Sculpture of gena: dorsoventrally strigose. Basiconic sensillum on A7: present.

Wings: brachypterous, apex of forewing ending before T4. Notaulus: percurrent. Color of mesosoma: variably orange to brown. Sculpture of mesoscutum medially: densely punctate, with longitudinal rugae in posterior half. Sculpture of mesoscutellum: smooth along midline, otherwise punctate rugulose. Dark bristlelike setae along transverse pronotal carina: absent. Sculpture ventral of transverse pronotal carina: finely punctate. Sculpture of femoral groove: striate in ventral end. Sculpture of ventral half of posterior mesepimeral area: smooth. Fine setigerous punctures on dorsal half of posterior mesepimeral area: present. Mesopleural carina: present along anterior half of femoral groove. Setation of ventral metapleural area: absent in area immediately below metapleural sulcus. Setation of metapleural triangle: dense. Color of legs: pale brown throughout.

Color of metasoma: orange to brown. Horn of T1: bulge smooth, at least anteriorly. Microsculpture of T2: absent. Microsculpture on T3: absent. Macrosculpture of T3 medially: absent. Macrosculpture of T3 laterally: longitudinally strigose. Microsculpture of T4: absent. Macrosculpture of T4 laterally: absent. Punctation of T4: moderately dense throughout. Macrosculpture of T5: absent. Punctation of T5: moderately dense throughout. Microsculpture of S3: absent. Macrosculpture of S3 laterally: longitudinally strigose.

##### Diagnosis.

 *Paridris ferus* and *Paridris reptilis* are the only brachypterous species known in the *Paridris nephta* group. Aside from this character, these two species are not particularly similar and may be separated by the presence of a basiconic sensillum on A7, the smooth form of the metapleural sulcus and longitudinal striation of S3 in *Paridris ferus*.

##### Etymology.

 The adjectival epithet “ferus” means “wild” or “untamed” in Latin and refers to the “savage” appearance of this species.

##### Link to Distribution Map.

^17^

##### Material Examined.

Holotype, female: **THAILAND:** Chiang Mai Prov., summit forest, T178, Doi Inthanon National Park, 18°35.361'N, 98°29.157'E , 2500m, 9.VIII–16.VIII.2006, malaise trap, Y. Areeluck, OSUC 192426 (deposited in QSBG).

#### Paridris kagemono

Talamas sp. n.

urn:lsid:zoobank.org:act:59410F97-5DD7-4E7E-A7C3-2F25DD7665AE

urn:lsid:biosci.ohio-state.edu:osuc_concepts:273916

http://species-id.net/wiki/Paridris_kagemono

**Figures 37–42.**
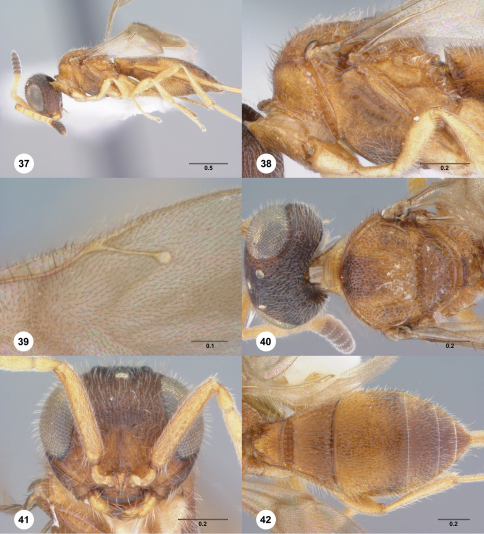
**^68^***Paridris kagemono* sp. n., female holotype (OSUC 262193) **37** Lateral habitus**38** Mesosoma, lateral view**39** Forewing, dorsal view**40** Head and mesosoma, dorsal view**41** Head, anterior view**42** Metasoma, dorsal view. Scale bars in millimeters.

Figures 37–42 Morphbank^18^ 

##### Description.

Female body length: 2.65 mm (n=1). Color of head: dark orange, becoming brown at vertex. Ventral clypeal margin: serrate. Sculpture of frons medially: dorsoventrally striate. Sculpture of frons immediately ventral of median ocellus: dorsoventrally striate throughout. Microsculpture of frons: present. Sculpture of posterior vertex: punctate rugulose. Sculpture of gena: irregularly rugulose. Basiconic sensillum on A7: absent.

Wings: macropterous, apex of forewing extending beyond posterior margin of T3. Length of R1: less than r-rs. Notaulus: percurrent. Color of mesosoma: orange throughout. Sculpture of mesoscutum medially: densely punctate throughout. Sculpture of mesoscutellum: punctate rugulose throughout. Dark bristlelike setae along transverse pronotal carina: absent. Sculpture ventral of transverse pronotal carina: punctate rugulose. Sculpture of femoral groove: striate below mesopleural pit. Sculpture of ventral half of posterior mesepimeral area: smooth. Fine setigerous punctures on dorsal half of posterior mesepimeral area: present. Mesopleural carina: absent. Setation of ventral metapleural area: absent in area immediately below metapleural sulcus. Setation of metapleural triangle: moderately dense. Color of legs: yellow throughout.

Color of metasoma: orange throughout. Horn of T1: absent. Microsculpture of T2: present. Microsculpture on T3: present. Macrosculpture of T3 medially: reticulate rugose. Macrosculpture of T3 laterally: reticulate rugose. Microsculpture of T4: absent. Macrosculpture of T4 laterally: weakly rugulose. Punctation of T4: moderately dense throughout. Macrosculpture of T5: absent. Punctation of T5: moderately dense throughout. Microsculpture of S3: absent. Macrosculpture of S3 laterally: absent.

##### Diagnosis.

 *Paridris kagemono* is most similar to *Paridris nephta*. It may be separated from it, and all other members of the *Paridris nephta* species group, by the presence of microsculpture between the striae of the frons.

##### Etymology.

 The epithet “kagemono” means “supernatural creature of the night” in Japanese. It is used as a noun in apposition.

##### Link to Distribution Map.

^19^

##### Material Examined.

Holotype, female: **JAPAN:** Fukuoka Pref., Kyushu Isl., primary evergreen forest, Mount Tachibana, 1.VII–6.VII.1979, yellow pan trap, K. Yamagishi, OSUC 262193 (deposited in CNCI).

#### Paridris minator

Talamas sp. n.

urn:lsid:zoobank.org:act:6A07840D-7470-4B42-89E2-7012B99C6C8F

urn:lsid:biosci.ohio-state.edu:osuc_concepts:241284

http://species-id.net/wiki/Paridris_minator

Figures 14 43–48 Morphbank^20^ 

##### Description.

Female body length: 2.27–2.53 mm (n=9). Color of head: uncertain, reddish brown. Ventral clypeal margin: serrate. Sculpture of frons medially: smooth. Sculpture of frons immediately ventral of median ocellus: dorsoventrally strigose throughout; rugose. Microsculpture of frons: absent. Sculpture of posterior vertex: finely punctate; punctate rugulose. Sculpture of gena: punctate rugulose; densely and finely punctate. Basiconic sensillum on A7: present.

Wings: macropterous, apex of forewing extending beyond posterior margin of T3. Length of R1: equal to r-rs; longer than r-rs. Notaulus: percurrent. Color of mesosoma: variably red to black. Sculpture of mesoscutum medially: densely punctate throughout. Sculpture of mesoscutellum: densely punctate. Dark bristlelike setae along transverse pronotal carina: absent. Sculpture ventral of transverse pronotal carina: finely punctate. Sculpture of femoral groove: smooth. Sculpture of ventral half of posterior mesepimeral area: smooth. Fine setigerous punctures on dorsal half of posterior mesepimeral area: present. Mesopleural carina: present. Setation of ventral metapleural area: absent in area immediately below metapleural sulcus. Setation of metapleural triangle: moderately dense. Color of legs: pale brown throughout; yellow throughout.

Color of metasoma: dark brown to black throughout; reddish brown. Horn of T1: bulge smooth, at least anteriorly. Microsculpture of T2: absent. Microsculpture on T3: present. Macrosculpture of T3 medially: reticulate; longitudinally strigose; weakly longitudinally strigose. Macrosculpture of T3 laterally: longitudinally strigose. Microsculpture of T4: absent. Macrosculpture of T4 laterally: weakly rugulose; absent. Punctation of T4: moderately dense throughout. Macrosculpture of T5: absent. Punctation of T5: moderately dense throughout. Microsculpture of S3: absent. Macrosculpture of S3 laterally: absent.

**Figures 43–48.**
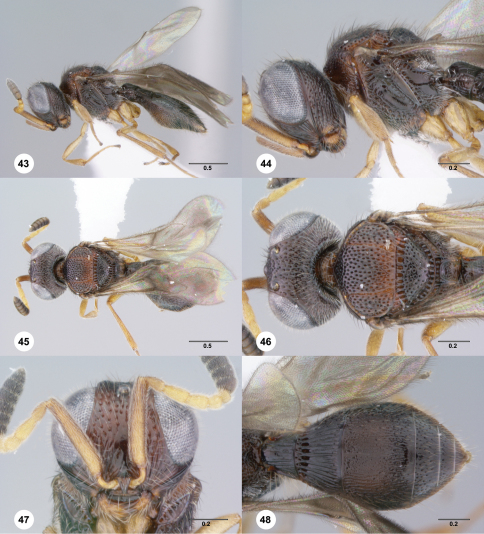
**^69^***Paridris minator* sp. n., female holotype (OSUC 237531) **43** Lateral habitus**44** Head and mesosoma, lateral view**45** Dorsal habitus**46** Head and mesosoma, dorsal view**47** Head, anterior view**48** Metasoma, dorsal view. Scale bars in millimeters.

##### Diagnosis.

 *Paridris minator* is similar to *Paridris solaris* in size, habitus and distribution and to *Paridris bunun* in its diagnostic characters. It is best separated from *Paridris solaris* by the presence of a basiconic sensillum on A7 and from *Paridris bunun* by the coarse punctation of the head and prominent striae of lateral T3.

##### Etymology.

 The Latin epithet “minator” means “threatener” and is given to this species for its fierce appearance.

##### Link to Distribution Map.

^21^

##### Material Examined.

 Holotype, female: **THAILAND:** Chiang Mai Prov., checkpoint 2, T73, Doi Inthanon National Park, 18°31.559'N, 98°29.941'E , 1700m, 15.VII–22.VII.2006, malaise trap, Y. Areeluck, OSUC 237531 (deposited in QSBG). *Paratypes*: (8 females) **LAOS:** 1 female, OSUC 334241 (CNCI). **THAILAND:** 7 females, OSUC 262239, 334245, 396845 (CNCI); OSUC 334205 (OSUC); OSUC 334005, 334215, 334246 (QSBG).

#### Paridris mystax

Talamas sp. n.

urn:lsid:zoobank.org:act:67E77FD7-4ECC-494F-B7B0-497E4B82AB59

urn:lsid:biosci.ohio-state.edu:osuc_concepts:241282

http://species-id.net/wiki/Paridris_mystax

**Figures 49–54.**
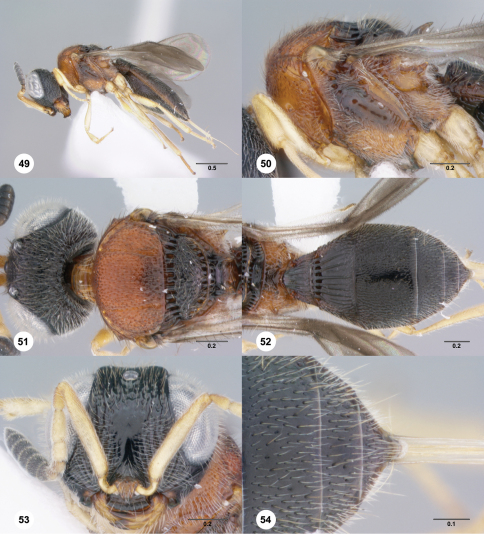
**^70^***Paridris mystax* sp. n. **49** Lateral habitus, female holotype (OSUC 237667)**50** Mesosoma, lateral view, female holotype (OSUC 237667)**51** Head and mesosoma, dorsal view, female holotype (OSUC 237667) **52** Metasoma, dorsal view, female (OSUC 262229)**53** Head, anterior view, female (OSUC 237533)**54** T4**–**T6, dorsal view, female holotype (OSUC 237667). Scale bars in millimeters.

Figures 49–54 111 Morphbank^22^ 

##### Description.

 Female body length: 2.53-3.26 mm (n=20). Color of head: black throughout. Ventral clypeal margin: serrate. Sculpture of frons medially: smooth. Sculpture of frons immediately ventral of median ocellus: dorsoventrally strigose throughout. Microsculpture of frons: absent. Sculpture of posterior vertex: punctate rugulose. Sculpture of gena: irregularly rugulose. Basiconic sensillum on A7: absent.

Wings: macropterous, apex of forewing extending beyond posterior margin of T3. Length of R1: equal to r-rs; less than r-rs. Notaulus: percurrent. Color of mesosoma: orange to dark red anteriorly, brown posteriorly, mesoscutellum black. Sculpture of mesoscutum medially: densely punctate throughout. Sculpture of mesoscutellum: punctate rugulose throughout; smooth along midline, otherwise punctate rugulose. Dark bristlelike setae along transverse pronotal carina: absent. Sculpture ventral of transverse pronotal carina: finely punctate. Sculpture of femoral groove: smooth; striate in ventral end. Sculpture of ventral half of posterior mesepimeral area: smooth. Fine setigerous punctures on dorsal half of posterior mesepimeral area: present. Mesopleural carina: present along anterior half of femoral groove. Setation of ventral metapleural area: present throughout. Setation of metapleural triangle: dense. Color of legs: yellow throughout.

Color of metasoma: orange to black. Horn of T1: bulge smooth, at least anteriorly. Microsculpture of T2: absent. Microsculpture on T3: present. Macrosculpture of T3 medially: reticulate; absent. Macrosculpture of T3 laterally: reticulate rugose. Microsculpture of T4: absent. Macrosculpture of T4 laterally: rugulose. Punctation of T4: dense throughout; moderately dense throughout. Macrosculpture of T5: weakly rugulose laterally; absent. Punctation of T5: dense throughout; moderately dense throughout. Microsculpture of S3: absent. Macrosculpture of S3 laterally: absent.

##### Diagnosis.

 *Paridris mystax* is one of the most distinctive species and can be easily identified by the dense setation throughout the ventral metapleural area and on the ventrolateral frons.

##### Etymology.

 The epithet “mystax”, meaning “hair on the upper lip” in Greek, is given to this species for the conspicuous setation of the ventral frons.

##### Link to Distribution Map.

^23^

##### Material Examined.

 Holotype, female: **THAILAND:** Loei Prov., Pong Neep Forest Unit, dry evergreen forest, T783, Phu Kradung National Park, 16°56.589'N, 101°42.074'E , 273m, 14.X–21.X.2006, malaise trap, S. Glong-lasae, OSUC 237667 (deposited in QSBG). *Paratypes*: (19 females) **LAOS:** 1 female, OSUC 265072 (CNCI). **THAILAND:** 18 females, OSUC 396840–396841, 396846 (CNCI); OSUC 254570, 254594–254595, 334210, 381817, 396837 (OSUC); OSUC 237533, 254569, 265198–265199, 334209, 334224–334226, 334228 (QSBG). *Other material*: **THAILAND:** 18 males, OSUC 181202, 181292, 237529, 396844, 396847 (CNCI); OSUC 254552, 265200, 334208, 334211, 334216 (OSUC); OSUC 237666, 261871, 265201, 266164, 334202–334203, 334212, 334227 (QSBG).

#### Paridris nephta

(Kozlov)

urn:lsid:zoobank.org:act:C8471114-0E15-44FB-BFA4-2FE3A0E7EAE8

urn:lsid:biosci.ohio-state.edu:osuc_concepts:243854

http://species-id.net/wiki/Paridris_nephta

**Figures 55–60.**
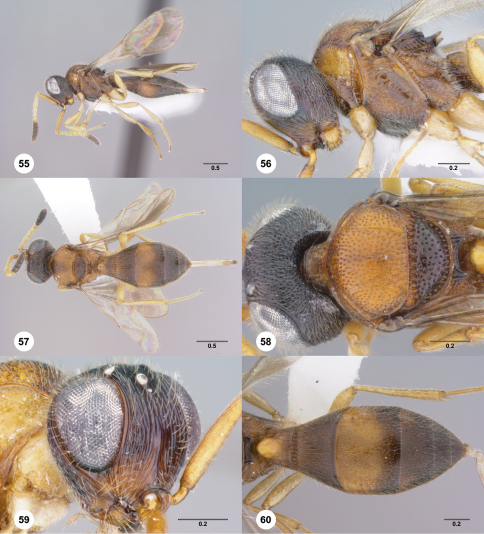
**^71^***Paridris nephta* **55** Lateral habitus, female (OSUC 265087)**56** Head and mesosoma, lateral view, female (OSUC 265087)**57** Dorsal habitus, female (OSUC 265150). 58, Head and mesosoma, dorsal view, female (OSUC 262229)**59** Head, anterolateral view, female (OSUC 143437)**60** Metasoma, dorsal view, female (OSUC 265195). Scale bars in millimeters.

Figures 55–60 Morphbank^24^

Tuora nephta  Kozlov, 1976: 98 (original description); Kozlov & Kononova, 1990: 263 (description of male and female); Kononova, 1995: 86 (keyed).
Paridris nephta  (Kozlov): Kononova & Kozlov, 2008: 279, 281 (description, keyed, generic transfer).

##### Description.

**Description.** Female body length: 2.53–3.10 mm (n=20). Color of head: dark brown to black. Ventral clypeal margin: serrate. Sculpture of frons medially: dorsoventrally striate. Sculpture of frons immediately ventral of median ocellus: dorsoventrally striate throughout. Microsculpture of frons: absent. Sculpture of posterior vertex: densely punctate. Sculpture of gena: finely punctate strigose; irregularly rugulose. Basiconic sensillum on A7: absent.

Wings: macropterous, apex of forewing extending beyond posterior margin of T3. Length of R1: equal to r-rs; less than r-rs. Notaulus: percurrent. Color of mesosoma: variably orange to brown. Sculpture of mesoscutum medially: densely punctate throughout. Sculpture of mesoscutellum: densely punctate. Dark bristlelike setae along transverse pronotal carina: absent. Sculpture ventral of transverse pronotal carina: finely punctate. Sculpture of femoral groove: striate below mesopleural pit. Sculpture of ventral half of posterior mesepimeral area: smooth. Fine setigerous punctures on dorsal half of posterior mesepimeral area: present. Mesopleural carina: present. Setation of ventral metapleural area: absent in area immediately below metapleural sulcus. Setation of metapleural triangle: sparse. Color of legs: yellow throughout.

Color of metasoma: orange to brown. Horn of T1: bulge smooth, at least anteriorly; present as a small bulge. Microsculpture of T2: present. Microsculpture on T3: present. Macrosculpture of T3 medially: reticulate rugose. Macrosculpture of T3 laterally: reticulate rugose. Microsculpture of T4: absent. Macrosculpture of T4 laterally: rugulose. Punctation of T4: dense throughout. Macrosculpture of T5: absent. Punctation of T5: dense throughout. Microsculpture of S3: absent. Macrosculpture of S3 laterally: absent; weakly longitudinally strigose.

##### Diagnosis.

 *Paridris nephta* is best distinguished by the uniform striation of the frons below the median ocellus, absence of microsculpture on the head and the smooth interspaces of the mesoscutellum. Color patterns are highly variable in this species and should be avoided entirely for identification.

##### Link to Distribution Map.

^25^

##### Material Examined.

 *Other material*: (129 females, 95 males) **JAPAN**: 73 females, 71 males, OSUC 181186–181189, 181191–181196, 181203–181209, 181213–181214, 181216–181218, 181221, 262145–262186, 262194–262199, 262225–262233, 262240–262247, 265063–265068, 265070–265071, 265073–265085, 265087–265089, 265093–265099, 265122–265132, 265134, 265139–265145, 265150–265152, 265155, 265195–265196 (CNCI). **RUSSIA:** 3 females, 3 males, OSUC 143437, 241513, 241655–241657, 404916 (OSUC). **SOUTH KOREA:** 53 females, 21 males, OSUC 181190, 181197, 181210, 181215, 181222–181225, 262187–262192, 262210–262219, 262221–262224, 262234, 262248–262258, 262260–262262, 265069, 265100–265121, 265136–265138, 265146–265149, 265197 (CNCI).

#### Paridris nilaka

Talamas sp. n.

urn:lsid:zoobank.org:act:EC0F912C-9AA2-4401-9251-4906A7B2BD1A

urn:lsid:biosci.ohio-state.edu:osuc_concepts:273890

http://species-id.net/wiki/Paridris_nilaka

Figures 13 61–66 109 Morphbank^26^ 

##### Description.

Female body length: 2.60–4.00 mm (n=7). Color of head: black throughout. Ventral clypeal margin: serrate. Sculpture of frons medially: smooth. Sculpture of frons immediately ventral of median ocellus: dorsoventrally strigose throughout; rugose. Microsculpture of frons: absent. Sculpture of posterior vertex: punctate rugulose. Sculpture of gena: punctate rugulose. Basiconic sensillum on A7: absent.

Wings: macropterous, apex of forewing extending beyond posterior margin of T3. Length of R1: less than r-rs. Notaulus: percurrent. Color of mesosoma: dark brown to black. Sculpture of mesoscutum medially: densely punctate, with longitudinal rugae in posterior half; areolate rugulose. Sculpture of mesoscutellum: punctate rugulose throughout. Dark bristle-like setae along transverse pronotal carina: absent. Sculpture ventral of transverse pronotal carina: finely punctate. Sculpture of femoral groove: smooth; striate in ventral end. Sculpture of ventral half of posterior mesepimeral area: smooth. Fine setigerous punctures on dorsal half of posterior mesepimeral area: present. Mesopleural carina: present along anterior half of femoral groove. Setation of ventral metapleural area: absent in area immediately below metapleural sulcus. Setation of metapleural triangle: dense. Color of legs: yellow throughout.

Color of metasoma: dark brown to black throughout. Horn of T1: bulge smooth, at least anteriorly; absent. Microsculpture of T2: absent. Microsculpture on T3: present. Macrosculpture of T3 medially: weakly longitudinally strigose. Macrosculpture of T3 laterally: reticulate rugose; longitudinally strigose. Microsculpture of T4: absent. Macrosculpture of T4 laterally: rugulose. Punctation of T4: sparse along midline, otherwise dense; dense throughout; moderately dense throughout. Macrosculpture of T5: absent; rugulose laterally. Punctation of T5: dense throughout; sparse medially, dense laterally. Microsculpture of S3: absent. Macrosculpture of S3 laterally: absent.

**Figures 61–66.**
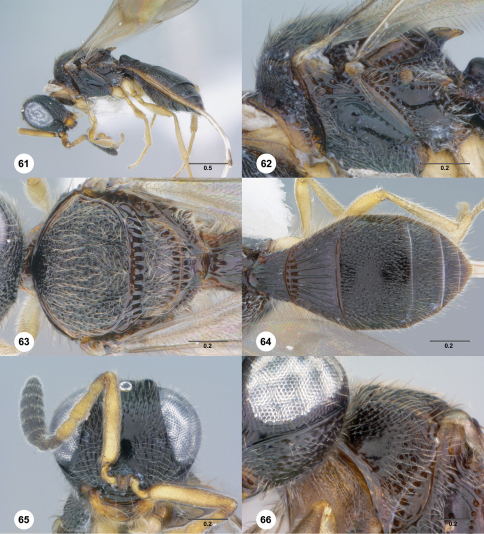
**^72^***Paridris nilaka* sp. n. **61** Lateral habitus, female holotype (OSUC 266165)**62** Mesosoma, lateral view, female holotype (OSUC 266165)**63** Mesosoma, dorsal view, female (OSUC 254613). 64, Metasoma, dorsal view, female holotype (OSUC 266165)**65** Head, anterior view, female holotype (OSUC 266165)**66** Pronotum, anterolateral view, female (OSUC 334223). Scale bars in millimeters.

##### Diagnosis.

 The rugulose sculpture of the dorsal mesoscutum and mesoscutellum in *Paridris nilaka* is shared with *Paridris rugulosus* and *Paridris toketoki*; it may be separated from both by the dense, fine setation of the pronotal shoulder. Additionally, the typically black color of the body may be useful for identification, but should be used with caution given the color plasticity seen in many species.

##### Etymology.

 The epithet “nilaka” means “black” in Thai, and is used as a noun in apposition.

##### Link to Distribution Map.

^27^ 

##### Material Examined.

 Holotype, female: **THAILAND:** Chiang Mai Prov., checkpoint 2, T1909, Doi Inthanon National Park, 18°31.554'N, 98°29.940'E , 1700m, 14.XI–15.XI.2006, pan trap, Y. Areeluck, OSUC 266165 (deposited in QSBG). *Paratypes*: **THAILAND:** 6 females, OSUC 334247 (CNCI); OSUC 254613, 381811 (OSUC); OSUC 334223, 334295, 396843 (QSBG).

#### Paridris reptilis

Talamas sp. n.

urn:lsid:zoobank.org:act:519CC4FD-EC66-48EA-9652-ECB6FDF14FC9

urn:lsid:biosci.ohio-state.edu:osuc_concepts:273878

http://species-id.net/wiki/Paridris_reptilis

**Figures 67–72.**
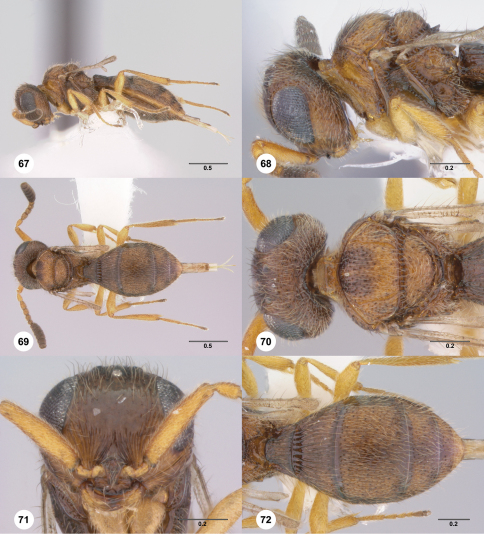
**^73^***Paridris reptilis* sp. n., female holotype (OSUC 181211) **67** Lateral habitus**68** Head and mesosoma, lateral view**69** Dorsal habitus**70** Head and mesosoma, dorsal view**71** Head, anterior view**72** Metasoma, dorsal view. Scale bars in millimeters.

Figures 67–72 Morphbank^28^ 

##### Description.

 Female body length: 2.35–2.40 mm (n=2). Color of head: reddish brown. Ventral clypeal margin: serrate. Sculpture of frons medially: mostly smooth with faint dorsoventral striation. Sculpture of frons immediately ventral of median ocellus: dorsoventrally strigose laterally. Microsculpture of frons: absent. Sculpture of posterior vertex: irregularly rugulose. Sculpture of gena: irregularly rugulose. Basiconic sensillum on A7: absent.

Wings: brachypterous, apex of forewing ending before T4. Notaulus: percurrent. Color of mesosoma: variably yellow to brown. Sculpture of mesoscutum medially: areolate rugulose. Sculpture of mesoscutellum: areolate rugulose. Dark bristlelike setae along transverse pronotal carina: absent. Sculpture ventral of transverse pronotal carina: rugulose posteriorly. Sculpture of femoral groove: striate in ventral end. Sculpture of ventral half of posterior mesepimeral area: smooth. Fine setigerous punctures on dorsal half of posterior mesepimeral area: present. Mesopleural carina: present. Setation of ventral metapleural area: absent in area immediately below metapleural sulcus. Setation of metapleural triangle: moderately dense. Color of legs: yellow throughout.

Color of metasoma: reddish brown. Horn of T1: bulge smooth, at least anteriorly; absent. Microsculpture of T2: present. Microsculpture on T3: absent. Macrosculpture of T3 medially: longitudinally strigose. Macrosculpture of T3 laterally: longitudinally strigose. Microsculpture of T4: absent. Macrosculpture of T4 laterally: rugulose. Punctation of T4: sparse along midline, otherwise dense. Macrosculpture of T5: absent. Punctation of T5: moderately dense throughout. Microsculpture of S3: absent. Macrosculpture of S3 laterally: absent.

##### Diagnosis.

 *Paridris reptilis* and *Paridris ferus* are the only known brachypterous species in the *Paridris nephta* group. *Paridris ferus* has a basiconic sensillum on A7 and lacks interstitial microsculpture on T2. *Paridris reptilis* does not have a sensillum on A7 and T2 is densely microsculptured.

##### Etymology.

 The adjectival epithet “reptilis”, meaning “crawling” in Latin, refers to the reduced wing size in this species.

##### Link to Distribution Map.

^29^

##### Material Examined.

 Holotype, female: **TAIWAN:** Taiwan Prov., Pingtung Co., Kuai-Ku Hut, T-103, Pei-ta-wu (Peitawushan) Mountain, 2125m, 26.IV–30.IV.1992, A. Smetana, OSUC 181211 (deposited in CNCI). *Paratype*: **TAIWAN:** 1 female, OSUC 265153 (CNCI).

#### Paridris rugulosus

Talamas sp. n.

urn:lsid:zoobank.org:act:8B3B767C-6BC7-40D8-B531-9DD8ED1B57EF

urn:lsid:biosci.ohio-state.edu:osuc_concepts:273914

http://species-id.net/wiki/Paridris_rugulosus

**Figures 73–78.**
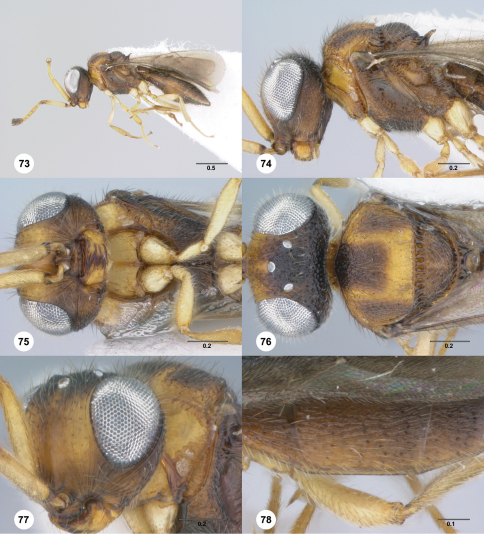
**^74^***Paridris rugulosus* sp. n., female holotype (OSUC 265238) **73** Lateral habitus**74** Head and mesosoma, lateral view**75** Head and mesosoma, ventral view**76** Head and mesosoma, dorsal view**77** Head and pronotum, anterolateral view**78** T2–T4, dorsolateral view. Scale bars in millimeters.

Figures 73–78 Morphbank^30^ 

##### Description.

 Female body length: 2.48–2.56 mm (n=2). Color of head: yellow, becoming darker dorsally; black throughout. Ventral clypeal margin: serrate. Sculpture of frons medially: smooth. Sculpture of frons immediately ventral of median ocellus: dorsoventrally strigose laterally; dorsoventrally strigose throughout. Microsculpture of frons: absent. Sculpture of posterior vertex: irregularly rugulose; punctate rugulose. Sculpture of gena: irregularly rugulose. Basiconic sensillum on A7: absent.

Wings: macropterous, apex of forewing extending beyond posterior margin of T3. Length of R1: equal to r-rs. Notaulus: percurrent. Color of mesosoma: variably yellow to brown. Sculpture of mesoscutum medially: areolate rugulose. Sculpture of mesoscutellum: punctate rugulose throughout; areolate rugulose. Dark bristlelike setae along transverse pronotal carina: present. Sculpture ventral of transverse pronotal carina: smooth. Sculpture of femoral groove: smooth; striate in ventral end. Sculpture of ventral half of posterior mesepimeral area: smooth. Fine setigerous punctures on dorsal half of posterior mesepimeral area: absent. Mesopleural carina: present. Setation of ventral metapleural area: absent in area immediately below metapleural sulcus. Setation of metapleural triangle: sparse. Color of legs: yellow throughout.

Color of metasoma: reddish brown. Horn of T1: absent. Microsculpture of T2: absent. Microsculpture on T3: absent. Macrosculpture of T3 medially: weakly longitudinally strigose. Macrosculpture of T3 laterally: longitudinally strigose. Microsculpture of T4: absent. Macrosculpture of T4 laterally: weakly rugulose. Punctation of T4: moderately dense throughout. Macrosculpture of T5: absent. Punctation of T5: moderately dense throughout. Microsculpture of S3: absent. Macrosculpture of S3 laterally: absent.

##### Diagnosis.

 *Paridris rugulosus* is most similar to *Paridris toketoki* and may be separated by the smooth surface of the lateral pronotum.

##### Etymology.

 The Latin adjectival epithet “rugulosus” refers to the rugulose sculpture of the head and dorsal mesosoma in this species.

##### Link to Distribution Map.

^31^

##### Material Examined.

 Holotype, female: **VIETNAM:** Vinh Phuc Prov., Tam Dao, 1050–1175m, 14.VI–17.VI.2007, malaise trap, C. v. Achterberg & R. de Vries, OSUC 265238 (deposited in RMNH). *Paratype*: **LAOS:** 1 female, OSUC 262200 (CNCI).

#### Paridris solaris

Talamas sp. n.

urn:lsid:zoobank.org:act:B2878DA2-1E8F-4B6E-96C4-86B571C11F04

urn:lsid:biosci.ohio-state.edu:osuc_concepts:241280

http://species-id.net/wiki/Paridris_solaris

**Figures 79–84.**
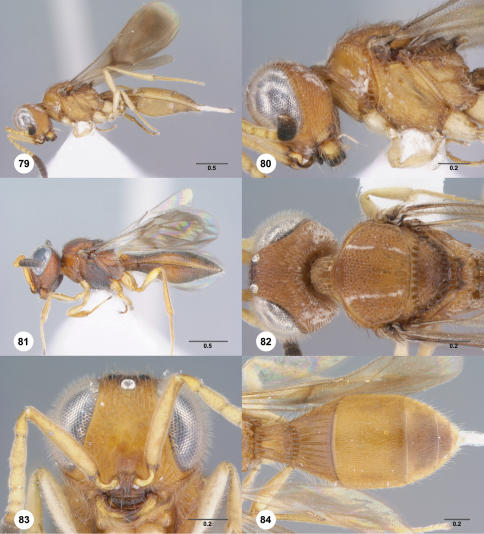
**^75^***Paridris solaris* sp. n. **79** Lateral habitus, female holotype (OSUC 240944)**80** Head and mesosoma, lateral view, female holotype (OSUC 240944)**81** Lateral habitus, female (OSUC 237532). 82, Head and mesosoma, dorsal view, female holotype (OSUC 240944)**83** Head, anterior view, female (OSUC 240948)**84** Metasoma, dorsal view, female (OSUC 240946). Scale bars in millimeters.

Figures 79–84 110 Morphbank^32^ 

##### Description.

 Female body length: 1.96–3.43 mm (n=19). Color of head: reddish brown; orange throughout; dark brown to black; yellow. Ventral clypeal margin: serrate. Sculpture of frons medially: smooth. Sculpture of frons immediately ventral of median ocellus: dorsoventrally strigose throughout; rugose. Microsculpture of frons: absent. Sculpture of posterior vertex: finely punctate; moderately punctate; punctate rugulose. Sculpture of gena: punctate rugulose. Basiconic sensillum on A7: absent.

Wings: macropterous, apex of forewing extending beyond posterior margin of T3. Length of R1: equal to r-rs; longer than r-rs. Notaulus: percurrent. Color of mesosoma: variably orange to brown; yellow throughout; orange throughout. Sculpture of mesoscutum medially: densely punctate, with longitudinal rugae in posterior half; densely punctate throughout. Sculpture of mesoscutellum: smooth medially, moderately punctate laterally; densely punctate. Dark bristlelike setae along transverse pronotal carina: absent. Sculpture ventral of transverse pronotal carina: finely punctate. Sculpture of femoral groove: smooth; striate in ventral end. Sculpture of ventral half of posterior mesepimeral area: smooth. Fine setigerous punctures on dorsal half of posterior mesepimeral area: present. Mesopleural carina: present. Setation of ventral metapleural area: absent in area immediately below metapleural sulcus. Setation of metapleural triangle: dense; moderately dense; sparse. Color of legs: yellow throughout.

Color of metasoma: yellow; orange to brown. Horn of T1: bulge smooth, at least anteriorly; absent. Microsculpture of T2: absent. Microsculpture on T3: absent. Macrosculpture of T3 medially: longitudinally strigose; weakly longitudinally strigose. Macrosculpture of T3 laterally: longitudinally strigose. Microsculpture of T4: absent. Macrosculpture of T4 laterally: absent. Punctation of T4: dense throughout. Macrosculpture of T5: absent. Punctation of T5: dense throughout; moderately dense throughout. Microsculpture of S3: absent. Macrosculpture of S3 laterally: weakly longitudinally strigose.

##### Diagnosis.

 *Paridris solaris* is most similar to *Paridris minator*. It may be separated from it by the absence of a basiconic sensillum on A7.

##### Etymology.

 The adjectival epithet “solaris” means “of the sun” in Latin and references the bright yellow-orange color present in many individuals of this species.

##### Link to Distribution Map.

^33^

##### Material Examined.

 Holotype, female: **VIETNAM:** Thua Thien-Hue Prov., ~1.5km NE along trail behind upper guesthouse, light gap / semi-tropical evergreen forest, ROM 2000512, Bach Ma National Park, 16°11'50.3"N, 107°51'17.7"E, 1200m, 6.VI–17.VI.2000, malaise trap/pan trap, B. Hubley, OSUC 240944 (deposited in ROME). *Paratypes*: (21 females) **LAOS:** 3 females, OSUC 334242–334243, 334248 (CNCI). **THAILAND:** 5 females, OSUC 334144, 396849 (OSUC); OSUC 237532, 265212, 334207 (QSBG). **VIETNAM:** 13 females, OSUC 240940 (IEBR); OSUC 240945, 404917–404918 (OSUC); OSUC 265234–265236, 277369, 281520 (RMNH); OSUC 240946, 240948, 266180, 404919 (ROME).

##### Comments.

 The color of specimens of *Paridris solaris* varies significantly according to geographical location. Those from Vietnam are typically yellow throughout (Fig. 79) and those from Thailand are variably orange, red, and black (Fig. 81).

#### Paridris teres

Talamas sp. n.

urn:lsid:zoobank.org:act:8A24D695-8FC0-4921-8885-B8FC3493FBE3

urn:lsid:biosci.ohio-state.edu:osuc_concepts:273893

http://species-id.net/wiki/Paridris_teres

**Figures 85–90.**
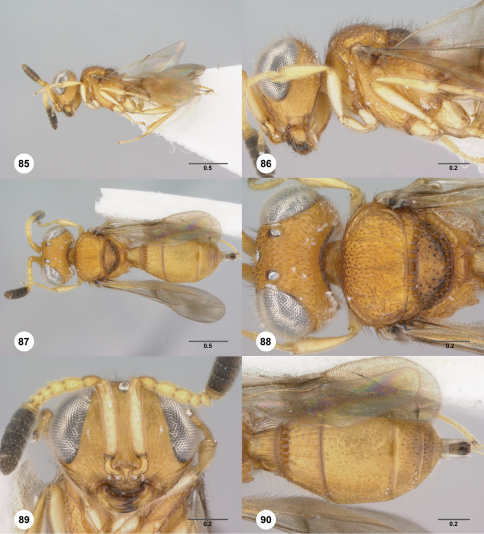
**^76^***Paridris teres* sp. n., female holotype (OSUC 265237) **85** Lateral habitus**86** Head and mesosoma, lateral view**87** Dorsal habitus**88** Head and mesosoma, dorsal view**89** Head, anterior view**90** Metasoma, dorsal view. Scale bars in millimeters.

Figures 85–90 Morphbank^34^ 

##### Description.

 Female body length: 2.42 mm (n=1). Color of head: yellow. Ventral clypeal margin: smooth. Sculpture of frons medially: smooth. Sculpture of frons immediately ventral of median ocellus: dorsoventrally strigose throughout. Microsculpture of frons: absent. Sculpture of posterior vertex: punctate rugulose. Sculpture of gena: punctate rugulose. Basiconic sensillum on A7: absent.

Wings: macropterous, apex of forewing extending beyond posterior margin of T3. Length of R1: equal to r-rs. Notaulus: percurrent. Color of mesosoma: yellow throughout. Sculpture of mesoscutum medially: densely punctate, with longitudinal rugae in posterior half. Sculpture of mesoscutellum: smooth along midline, otherwise punctate rugulose. Dark bristlelike setae along transverse pronotal carina: absent. Sculpture ventral of transverse pronotal carina: finely punctate. Sculpture of ventral half of posterior mesepimeral area: smooth. Fine setigerous punctures on dorsal half of posterior mesepimeral area: present. Mesopleural carina: present. Setation of ventral metapleural area: absent in area immediately below metapleural sulcus. Setation of metapleural triangle: moderately dense. Color of legs: yellow throughout.

Color of metasoma: yellow. Horn of T1: absent. Microsculpture of T2: absent. Microsculpture on T3: present. Macrosculpture of T3 medially: absent. Macrosculpture of T3 laterally: absent. Microsculpture of T4: absent. Macrosculpture of T4 laterally: weakly rugulose. Punctation of T4: dense throughout. Macrosculpture of T5: rugulose laterally. Punctation of T5: moderately dense throughout. Microsculpture of S3: absent. Macrosculpture of S3 laterally: weakly longitudinally strigose.

##### Diagnosis.

 *Paridris teres* may be easily identified by the smooth ventral margin of the clypeus.

##### Etymology.

 The epithet “teres”, meaning smooth in Latin, refers to the smooth margin of the clypeus and is used as a noun in apposition.

##### Link to Distribution Map.

^35^

##### Material Examined.

Holotype, female: **VIETNAM:** Vinh Phuc Prov., Tam Dao, 1050–1175m, 14.VI–17.VI.2007, malaise trap, C. v. Achterberg & R. de Vries, OSUC 265237 (deposited in RMNH).

##### Comments.

The sole specimen of this species was damaged during examination after it was imaged. The head, propleuron and forelegs are now mounted on the point separate from the remainder of the body; A7–12 of the right antenna are lost.

#### Paridris toketoki

Talamas sp. n.

urn:lsid:zoobank.org:act:628BBEF3-C3BA-4EE4-A905-3334CBD8ED7F

urn:lsid:biosci.ohio-state.edu:osuc_concepts:273915

http://species-id.net/wiki/Paridris_toketoki

**Figures 91–96.**
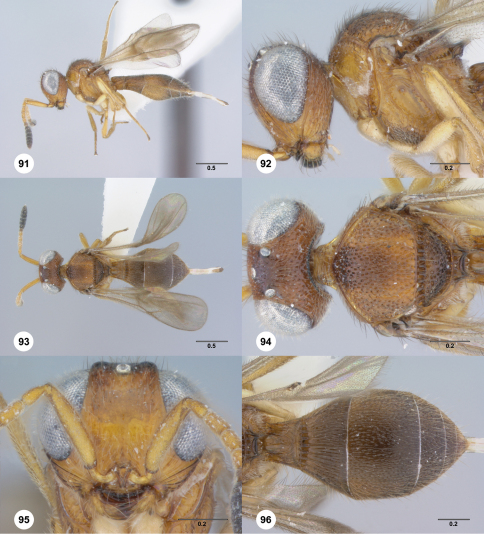
**^77^***Paridris toketoki* sp. n., female holotype (OSUC 181200) **91** Lateral habitus**92** Head and mesosoma, lateral view**93** Dorsal habitus**94** Head and mesosoma, dorsal view**95** Head, anterior view**96** Metasoma, dorsal view. Scale bars in millimeters.

Figures 91–96 Morphbank^36^ 

##### Description.

 Female body length: 2.54 mm (n=1). Color of head: dark orange, becoming brown at vertex. Ventral clypeal margin: serrate. Sculpture of frons medially: smooth. Sculpture of frons immediately ventral of median ocellus: dorsoventrally strigose laterally. Microsculpture of frons: absent. Sculpture of posterior vertex: punctate rugulose. Sculpture of gena: punctate rugulose. Basiconic sensillum on A7: absent.

Wings: macropterous, apex of forewing extending beyond posterior margin of T3. Length of R1: less than r-rs. Notaulus: smooth furrow incomplete, reaching suprahumeral sulcus as row of punctures. Color of mesosoma: variably orange to brown. Sculpture of mesoscutum medially: densely punctate throughout. Sculpture of mesoscutellum: punctate rugulose throughout. Dark bristlelike setae along transverse pronotal carina: present. Sculpture ventral of transverse pronotal carina: finely punctate. Sculpture of femoral groove: smooth. Sculpture of ventral half of posterior mesepimeral area: smooth. Fine setigerous punctures on dorsal half of posterior mesepimeral area: present. Mesopleural carina: present along anterior half of femoral groove. Setation of ventral metapleural area: absent in area immediately below metapleural sulcus. Setation of metapleural triangle: moderately dense. Color of legs: yellow throughout.

Color of metasoma: orange to brown. Horn of T1: bulge smooth, at least anteriorly. Microsculpture of T2: absent. Microsculpture on T3: uncertain, absent. Macrosculpture of T3 medially: weakly longitudinally strigose. Macrosculpture of T3 laterally: longitudinally rugulose. Microsculpture of T4: absent. Macrosculpture of T4 laterally: rugulose. Punctation of T4: dense throughout. Macrosculpture of T5: rugulose laterally. Punctation of T5: sparse medially, dense laterally. Microsculpture of S3: absent. Macrosculpture of S3 laterally: weakly longitudinally strigose.

##### Diagnosis.

 *Paridris toketoki* is most similar to *Paridris rugulosus*. It differs most conspicuously in having the lateral face of the pronotum densely punctate along its dorsal margin.

##### Etymology.

 This species is named for the great Paiwan chief, Toketok.

##### Link to Distribution Map.

^37^

##### Material Examined.

Holotype, female: **TAIWAN:** Taiwan Prov., Nantou Co., Jih-yüeh (Sun Moon) Lake, H025, Te-hua-she (Tehuache), 800m, 5.VI.1980, J. Heraty, OSUC 181200 (deposited in CNCI).

#### Paridris verrucosus

Talamas sp. n.

urn:lsid:zoobank.org:act:CCEB3258-CADF-4F0F-B2E2-983F94AF5372

urn:lsid:biosci.ohio-state.edu:osuc_concepts:275741

http://species-id.net/wiki/Paridris_verrucosus

Figures 17 97–102 Morphbank^38^ 

##### Description.

 Female body length: 1.97 mm (n=1). Color of head: dark brown to black. Ventral clypeal margin: serrate. Sculpture of frons medially: smooth. Sculpture of frons immediately ventral of median ocellus: dorsoventrally strigose throughout. Microsculpture of frons: uncertain, absent. Sculpture of posterior vertex: irregularly rugulose. Sculpture of gena: irregularly rugulose. Basiconic sensillum on A7: absent.

Wings: macropterous, apex of forewing extending beyond posterior margin of T3. Length of R1: equal to r-rs. Notaulus: absent. Color of mesosoma: variably orange to brown. Sculpture of mesoscutum medially: areolate rugulose. Sculpture of mesoscutellum: areolate rugulose. Dark bristlelike setae along transverse pronotal carina: present. Sculpture ventral of transverse pronotal carina: rugulose posteriorly. Sculpture of femoral groove: striate below mesopleural pit. Sculpture of ventral half of posterior mesepimeral area: rugulose. Fine setigerous punctures on dorsal half of posterior mesepimeral area: absent. Mesopleural carina: present. Setation of ventral metapleural area: absent in area immediately below metapleural sulcus. Setation of metapleural triangle: moderately dense. Color of legs: yellow throughout.

Color of metasoma: reddish brown. Horn of T1: absent. Microsculpture of T2: present. Microsculpture on T3: present. Macrosculpture of T3 medially: reticulate. Macrosculpture of T3 laterally: longitudinally rugulose. Microsculpture of T4: present. Macrosculpture of T4 laterally: rugulose. Punctation of T4: moderately dense throughout. Macrosculpture of T5: rugulose laterally. Punctation of T5: moderately dense laterally and along anterior margin. Microsculpture of S3: present. Macrosculpture of S3 laterally: longitudinally strigose.

**Figures 97–102.**
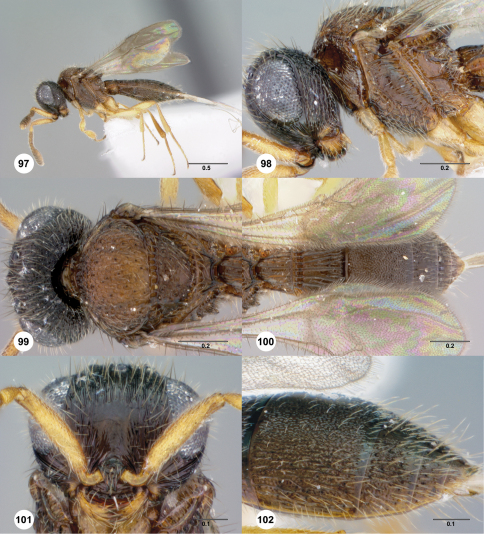
**^78^***Paridris verrucosus* sp. n., female holotype (OSUC 334249) **97** Lateral habitus**98** Head and mesosoma, lateral view**99** Head and mesosoma, dorsal view**100** Metasoma, dorsal view**101** Head, anterior view**102** T3–T6, dorsolateral view. Scale bars in millimeters.

##### Diagnosis.

 *Paridris verrucosus* is the only species in the *Paridris nephta* group with microsculpture on S3.

##### Etymology.

 The adjectival epithet “verrucosus” means “full of warts” in Latin; it is given to this species for the dense microsculpture of the metasoma.

##### Link to Distribution Map.

^39^

##### Material Examined.

Holotype, female: **CHINA:** Guangdong Prov., creek, Nankunshan, 23°37.287'N, 113°51.267'S , 581m, 29.X–31.X.2009, yellow pan trap, L. Masner, OSUC 334249 (deposited in CNCI).

#### Paridris yak

Talamas sp. n.

urn:lsid:zoobank.org:act:37D0E197-226E-4EB5-B367-76F0C5D46276

urn:lsid:biosci.ohio-state.edu:osuc_concepts:241283

http://species-id.net/wiki/Paridris_yak

Figures 15 103–108 Morphbank^40^ 

##### Description.

 Female body length: 4.15–4.16 mm (n=3). Color of head: dark orange, becoming brown at vertex. Ventral clypeal margin: serrate. Sculpture of frons medially: smooth. Sculpture of frons immediately ventral of median ocellus: rugose. Microsculpture of frons: absent. Sculpture of posterior vertex: areolate rugulose. Sculpture of gena: punctate rugulose. Basiconic sensillum on A7: absent.

Wings: macropterous, apex of forewing extending beyond posterior margin of T3. Length of R1: equal to r-rs; less than r-rs. Notaulus: absent; indicated only at posterior margin of mesoscutum. Color of mesosoma: orange to dark red anteriorly, brown posteriorly, mesoscutellum black; variably red to black. Sculpture of mesoscutum medially: areolate rugulose. Sculpture of mesoscutellum: areolate rugulose. Dark bristlelike setae along transverse pronotal carina: absent. Sculpture ventral of transverse pronotal carina: finely punctate. Sculpture of femoral groove: smooth. Sculpture of ventral half of posterior mesepimeral area: smooth. Fine setigerous punctures on dorsal half of posterior mesepimeral area: present. Mesopleural carina: present. Setation of ventral metapleural area: absent in area immediately below metapleural sulcus. Setation of metapleural triangle: moderately dense. Color of legs: yellow throughout.

Color of metasoma: orange to black. Horn of T1: bulge smooth, at least anteriorly. Microsculpture of T2: absent. Microsculpture on T3: absent. Macrosculpture of T3 medially: longitudinally strigose; weakly longitudinally strigose. Macrosculpture of T3 laterally: longitudinally strigose. Microsculpture of T4: absent. Macrosculpture of T4 laterally: rugulose. Punctation of T4: dense throughout. Macrosculpture of T5: rugulose laterally. Punctation of T5: dense throughout. Microsculpture of S3: absent. Macrosculpture of S3 laterally: absent.

**Figures 103–108.**
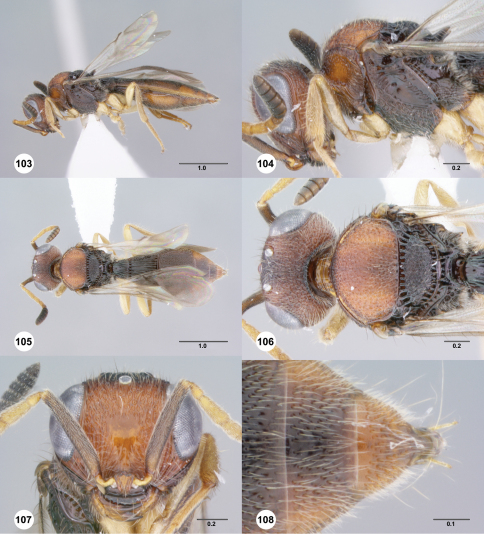
**^79^***Paridris yak* sp. n., female holotype (OSUC 237530) **103** Lateral habitus**104** Head and mesosoma, lateral view**105** Dorsal habitus**106** Head and mesosoma, dorsal view**107** Head, anterior view**108** T5**–**T6, dorsal view. Scale bars in millimeters.

**Figures 109–111.**
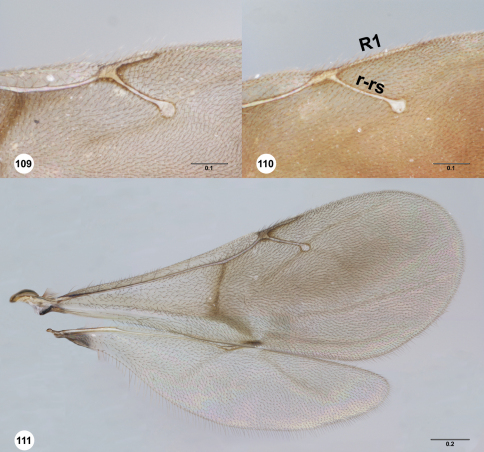
**^80^** **109** *Paridris nilaka* sp. n., R1 (postmarginal vein) and r-rs (stigmal vein), dorsal view, female holotype (OSUC 266165)**110** *Paridris solaris*, sp. n., R1 (postmarginal vein) and r-rs (stigmal vein), dorsal view, female holotype (OSUC 240944)**111** *Paridris mystax*, sp. n., fore and hind wing, dorsal view, male (OSUC 265200). Scale bars in millimeters.

##### Diagnosis.

 *Paridris yak* is a large distinctive species best identified by its reduced or absent notaulus, dorsally rugose frons and dorsally pointed axillular carina.

##### Etymology.

 The word “yak” is Thai for a mythological ogre. It is treated as a noun in apposition.

##### Link to Distribution Map.

^41^

##### Material Examined.

 Holotype, female: **THAILAND:** Trang Prov., forest research center, Khao Chong Mountain, 07°33.2'N, 99°47.22'E , 75m, XI–2005, malaise trap, D. Lohman, OSUC 237530 (deposited in QSBG). *Paratypes*: **THAILAND:** 3 females, OSUC 396848 (OSUC); OSUC 266085, 334214 (QSBG).

## Supplementary Material

XML Treatment for Paridris atrox

XML Treatment for Paridris bunun

XML Treatment for Paridris ferus

XML Treatment for Paridris kagemono

XML Treatment for Paridris minator

XML Treatment for Paridris mystax

XML Treatment for Paridris nephta

XML Treatment for Paridris nilaka

XML Treatment for Paridris reptilis

XML Treatment for Paridris rugulosus

XML Treatment for Paridris solaris

XML Treatment for Paridris teres

XML Treatment for Paridris toketoki

XML Treatment for Paridris verrucosus

XML Treatment for Paridris yak

## Figures and Tables

**Table:** Abbreviations Label Used in Text Unique Identyfier of HAO class Label Used in Talamas et al. 2011

axc	axillular carina	http://purl.obolibrary.org/obo/HAO_0000161	
	basiconic sensillum	http://purl.obolibrary.org/obo/HAO_0001729	
	body	http://purl.obolibrary.org/obo/HAO_0000182	
	compound eye	http://purl.obolibrary.org/obo/HAO_0000217	
	conjunctiva	http://purl.obolibrary.org/obo/HAO_0000221	
epc	epomial carina	http://purl.obolibrary.org/obo/HAO_0000307	vertical epomial carina
	felt field	http://purl.obolibrary.org/obo/HAO_0000322	
	femoral groove	http://purl.obolibrary.org/obo/HAO_0000326	femoral depresssion
	frons	http://purl.obolibrary.org/obo/HAO_0001044	
	gena	http://purl.obolibrary.org/obo/HAO_0000371	
	horn of T1		
	inner orbit of compound eye	http://purl.obolibrary.org/obo/HAO_0000419	
	interantennal process	http://purl.obolibrary.org/obo/HAO_0000422	
loc	lateral ocellus	http://purl.obolibrary.org/obo/HAO_0000481	
	lateral propodeal carina	http://purl.obolibrary.org/obo/HAO_0000486	
	leg	http://purl.obolibrary.org/obo/HAO_0000494	
	mandible	http://purl.obolibrary.org/obo/HAO_0000506	
	median ocellus	http://purl.obolibrary.org/obo/HAO_0000526	
	mesepisternum	http://purl.obolibrary.org/obo/HAO_0000541	
	mesoscutal suprahumeral sulcus	http://purl.obolibrary.org/obo/HAO_0000570	
	mesoscutellum	http://purl.obolibrary.org/obo/HAO_0000574	
	mesoscutum	http://purl.obolibrary.org/obo/HAO_0000575	
	mesosoma	http://purl.obolibrary.org/obo/HAO_0000576	
mtps	metapleural sulcus	http://purl.obolibrary.org/obo/HAO_0000614	
	metapleural triangle	http://purl.obolibrary.org/obo/HAO_0000615	
	metapleuron	http://purl.obolibrary.org/obo/HAO_0001869	
	metascutellum	http://purl.obolibrary.org/obo/HAO_0000625	
	metasoma	http://purl.obolibrary.org/obo/HAO_0000626	
	metasomal depression	http://purl.obolibrary.org/obo/HAO_0000627	
	notaulus	http://purl.obolibrary.org/obo/HAO_0000647	
	occipital carina	http://purl.obolibrary.org/obo/HAO_0000653	
	ovipositor	http://purl.obolibrary.org/obo/HAO_0000679	
pcxs	paracoxal sulcus	http://purl.obolibrary.org/obo/HAO_0000685	
	plica	http://purl.obolibrary.org/obo/HAO_0000735	
	posterior mesepimeral area	http://purl.obolibrary.org/obo/HAO_0000751	
	posterior scutellar sulcus	http://purl.obolibrary.org/obo/HAO_0000757	
	pronotum	http://purl.obolibrary.org/obo/HAO_0000853	
	posterior propodeal projection	http://purl.obolibrary.org/obo/HAO_0000763	
	propodeum	http://purl.obolibrary.org/obo/HAO_0001249	
	S2	http://purl.obolibrary.org/obo/HAO_0000035	
	scutoscutellar sulcus	http://purl.obolibrary.org/obo/HAO_0000920	
	T2	http://purl.obolibrary.org/obo/HAO_0001147	
	T3	http://purl.obolibrary.org/obo/HAO_0001151	
	T6	http://purl.obolibrary.org/obo/HAO_0001468	
trc	transverse carina on T2		
tpc	transverse pronotal carina	http://purl.obolibrary.org/obo/HAO_0001031	dorsal epomial carina
	ventral clypeal margin	http://purl.obolibrary.org/obo/HAO_0001767	
	ventral metapleural area	http://purl.obolibrary.org/obo/HAO_0001062	
	vertex	http://purl.obolibrary.org/obo/HAO_0001077	

## Endnotes

1	http://biocol.org/urn:lsid:biocol.org:col:1012

2	http://biocol.org/urn:lsid:biocol.org:col:35284

3	http://biocol.org/urn:lsid:biocol.org:col:33578

4	http://biocol.org/urn:lsid:biocol.org:col:1014

5	http://biocol.org/urn:lsid:biocol.org:col:1016

6	http://biocol.org/urn:lsid:biocol.org:col:1017

7	http://biocol.org/urn:lsid:biocol.org:col:34212

8	http://morphbank.net/?id=592382

9	http://morphbank.net/?id=592383

10	http://morphbank.net/?id=592384

11	http://morphbank.net/?id=592385

12	http://morphbank.net/?id=592369

13	http://hol.osu.edu/map-large.html?id=275737

14	http://morphbank.net/?id=592365

15	http://hol.osu.edu/map-large.html?id=273886

16	http://morphbank.net/?id=592344

17	http://hol.osu.edu/map-large.html?id=241281

18	http://morphbank.net/?id=592366

19	http://hol.osu.edu/map-large.html?id=273916

20	http://morphbank.net/?id=592362

21	http://hol.osu.edu/map-large.html?id=241284

22	http://morphbank.net/?id=592379

23	http://hol.osu.edu/map-large.html?id=241282

24	http://morphbank.net/?id=592377

25	http://hol.osu.edu/map-large.html?id=243854

26	http://morphbank.net/?id=592370

27	http://hol.osu.edu/map-large.html?id=273890

28	http://morphbank.net/?id=592367

29	http://hol.osu.edu/map-large.html?id=273878

30	http://morphbank.net/?id=592364

31	http://hol.osu.edu/map-large.html?id=273914

32	http://morphbank.net/?id=592363

33	http://hol.osu.edu/map-large.html?id=241280

34	http://morphbank.net/?id=592343

35	http://hol.osu.edu/map-large.html?id=273893

36	http://morphbank.net/?id=592374

37	http://hol.osu.edu/map-large.html?id=273915

38	http://morphbank.net/?id=592375

39	http://hol.osu.edu/map-large.html?id=275741

40	http://morphbank.net/?id=592368

41	http://hol.osu.edu/map-large.html?id=241283

42	http://lsid.tdwg.org/urn:lsid:biosci.ohio-state.edu:osuc_pubs:131

43	http://lsid.tdwg.org/urn:lsid:biosci.ohio-state.edu:osuc_pubs:330

44	http://lsid.tdwg.org/urn:lsid:biosci.ohio-state.edu:osuc_pubs:22163

45	http://lsid.tdwg.org/urn:lsid:biosci.ohio-state.edu:osuc_pubs:9730

46	http://lsid.tdwg.org/urn:lsid:biosci.ohio-state.edu:osuc_pubs:1501

47	http://lsid.tdwg.org/urn:lsid:biosci.ohio-state.edu:osuc_pubs:968

48	http://lsid.tdwg.org/urn:lsid:biosci.ohio-state.edu:osuc_pubs:340

49	http://lsid.tdwg.org/urn:lsid:biosci.ohio-state.edu:osuc_pubs:9718

50	http://lsid.tdwg.org/urn:lsid:biosci.ohio-state.edu:osuc_pubs:236

51	http://lsid.tdwg.org/urn:lsid:biosci.ohio-state.edu:osuc_pubs:311

52	http://lsid.tdwg.org/urn:lsid:biosci.ohio-state.edu:osuc_pubs:474

53	http://lsid.tdwg.org/urn:lsid:biosci.ohio-state.edu:osuc_pubs:312

54	http://lsid.tdwg.org/urn:lsid:biosci.ohio-state.edu:osuc_pubs:7039

55	http://www.mapress.com/zootaxa/2007f/zt01571p078.pdf

56	http://lsid.tdwg.org/urn:lsid:biosci.ohio-state.edu:osuc_pubs:21224

57	http://lsid.tdwg.org/urn:lsid:biosci.ohio-state.edu:osuc_pubs:20959

58	http://lsid.tdwg.org/urn:lsid:biosci.ohio-state.edu:osuc_pubs:21131

59	http://lsid.tdwg.org/urn:lsid:biosci.ohio-state.edu:osuc_pubs:23390

60	http://lsid.tdwg.org/urn:lsid:biosci.ohio-state.edu:osuc_pubs:3440

61	doi: 10.1371/journal.pone.0015991

62	http://morphbank.net/?id=592361

63	http://morphbank.net/?id=592381

64	http://morphbank.net/?id=592386

65	http://morphbank.net/?id=592387

66	http://morphbank.net/?id=592388

67	http://morphbank.net/?id=592373

68	http://morphbank.net/?id=592389

69	http://morphbank.net/?id=592390

70	http://morphbank.net/?id=592393

71	http://morphbank.net/?id=592394

72	http://morphbank.net/?id=592380

73	http://morphbank.net/?id=592395

74	http://morphbank.net/?id=592396

75	http://morphbank.net/?id=592397

76	http://morphbank.net/?id=592378

77	http://morphbank.net/?id=592398

78	http://morphbank.net/?id=592399

79	http://morphbank.net/?id=592400

80	http://morphbank.net/?id=592401

## Appendix II

Lucid key to females of the *Paridris nephta* species group. (doi: 10.3897/zookeys.133.1613.app)

Copyright notice: This dataset is made available under the Open Database License (http://opendatacommons.org/licenses/odbl/1.0/). The Open Database License (ODbL) is a license agreement intended to allow users to freely share, modify, and use this Dataset while maintaining this same freedom for others, provided that the original source and author(s) are credited.
